# *Leishmania guyanensis* suppressed inducible nitric oxide synthase provoked by its viral endosymbiont

**DOI:** 10.3389/fcimb.2022.944819

**Published:** 2022-08-12

**Authors:** Dmitry Kopelyanskiy, Chantal Desponds, Florence Prevel, Matteo Rossi, Romain Migliorini, Tiia Snäkä, Remzi Onur Eren, Stéphanie Claudinot, Lon-Fye Lye, Manolis Pasparakis, Stephen M. Beverley, Nicolas Fasel

**Affiliations:** ^1^ Department of Biochemistry, University of Lausanne, Epalinges, Switzerland; ^2^ Institute for Genetics, Cologne Excellence Cluster on Cellular Stress Responses in Aging-Associated Diseases (CECAD) and Center for Molecular Medicine, University of Cologne, Cologne, Germany; ^3^ Department of Molecular Microbiology, School of Medicine, Washington University, St. Louis, MO, United States

**Keywords:** *Leishmania*, *Leishmania* RNA virus 1 (LRV1), type I Interferons, nuclear factor kappa-light-chain-enhancer of activated B cells (NF-kB), inducible nitric oxide synthase (iNOS), tumor necrosis factor-alpha-induced protein 3 (A20), IL-17A, metastasis

## Abstract

Inducible nitric oxide synthase (iNOS) is essential to the production of nitric oxide (NO), an efficient effector molecule against intracellular human pathogens such as *Leishmania* protozoan parasites. Some strains of *Leishmania* are known to bear a viral endosymbiont termed *Leishmania* RNA virus 1 (LRV1). Recognition of LRV1 by the innate immune sensor Toll-like receptor-3 (TLR3) leads to conditions worsening the disease severity in mice. This process is governed by type I interferon (type I IFNs) arising downstream of TLR3 stimulation and favoring the formation of secondary metastatic lesions. The formation of these lesions is mediated by the inflammatory cytokine IL-17A and occurs in the absence, or low level of, protective cytokine IFN-γ. Here, we described that the presence of LRV1 led to the initial expression of iNOS and low production of NO that failed to control infection. We subsequently showed that LRV1-triggered type I IFN was essential but insufficient to induce robust iNOS induction, which requires strong activation of nuclear factor kappa-light-chain-enhancer of activated B cells (NF-κB). *Leishmania guyanensis* carrying LRV1 (*Lgy*LRV1+) parasites mitigated strong iNOS production by limiting NF-kB activation *via* the induction of tumor necrosis factor-alpha-induced protein 3 (TNFAIP3), also known as A20. Moreover, our data suggested that production of LRV1-induced iNOS could be correlated with parasite dissemination and metastasis *via* elevated secretion of IL-17A in the draining lymph nodes. Our findings support an additional strategy by which LRV1-bearing *Leishmania guyanensis* evaded killing by nitric oxide and suggest that low levels of LRV1-induced NO might contribute to parasite metastasis.

## Introduction

Inducible nitric oxide synthases (iNOS) is a member of a family of three enzymes responsible for conversion of L-arginine to L-citrulline, an important metabolic process accompanied by the release of nitric oxide (NO). Unlike endothelial NOS (eNOS) and neuronal NOS (nNOS), two constitutively expressed Ca2+ dependent isoforms, expression of iNOS does not require Ca2+ intake and does not occur in resting cells. Several inflammatory stimuli can trigger iNOS expression and the production of large amounts of NO. This molecule has many different functions and was described, amongst others, to have regulatory effects on angiogenesis, tumor metastasis and cell adhesion (Bogdan, 2015). Most importantly, NO plays a crucial role in the immune protection against intracellular pathogens as it is directly responsible for their elimination. Thus, iNOS is the key player mediating the immune response to many microbial infections.

Toll-like receptors (TLRs) are the most known and best-characterized family of pattern-recognition receptors (PRRs) (Akira et al., 2006). They play a crucial role in detection of pathogenic intruders. TLRs initiate microbial clearance and production of pro-inflammatory effector cytokines and chemokines that further direct the adaptive immune response (Majewska and Szczepanik, 2006). Most of the TLRs signal *via* the myeloid differentiation primary response protein 88 (MyD88), whilst TLR3 is the only TLR that acts solely *via* the TIR-inducing IFN-β (TRIF) molecule. TRIF signaling promotes an antiviral innate response *via* activation of the interferon regulatory factor 3 (IRF-3), which leads to the transcription of type I interferons (type I IFNs). Both MyD88 and TRIF signaling cascades lead to the nuclear translocation of the nuclear factor kappa-light-chain-enhancer of activated B cells (NF-κB) and activator protein-1 (AP-1) transcription factors.

NF-kB is the key player in inflammation and immunity. This complex encompasses five different members containing the Rel homology domain: NF-kB1 (p105/p50), NF-kB2 (p100/p52), RelA (p65), RelB and c-Rel (Hoffmann et al., 2006). The most abundant member is the p65p50 heterodimer (Hayden and Ghosh, 2012) which is usually associated with activation of transcription. This in turn leads to the consequent transcription of pro-inflammatory cytokines, such as tumor necrosis factor alpha (TNF-α) and Interleukin 6 (IL-6) (Kawai and Akira, 2006). As a major negative feedback regulator of NF-kB-mediated inflammation, tumor necrosis factor alpha-induced protein 3 (TNFAIP3) (also named A20) is induced very early in the NF-kB response (Coornaert et al., 2009; Catrysse et al., 2014).

*Leishmania* is a genus of protozoan parasites that exists in two morphological forms. The flagellated promastigote forms are transmitted by a sand fly vector to the mammalian hosts. Upon inoculation, parasites are engulfed by tissue resident macrophages, where they convert into the round amastigote form. *Leishmania* survive and proliferate inside the macrophages causing different types of pathologies depending on the infecting species and immune competence of the host. Termed leishmaniasis, this range of diseases affects over 12 million people in 98 countries worldwide (Kaye and Scott, 2011; Alvar et al., 2014). Mucocutaneous leishmaniasis (MCL) and disseminated cutaneous leishmaniasis (DCL) develop mainly due to infection with species of *Leishmania Viannia* subgenus, such as *Leishmania guyanensis* (*Lgy*) and *Leishmania brasiliensis* (*Lbr*). These pathologies arise as complications of cutaneous leishmaniasis (CL) and are characterized by metastatic lesions coming from primary sites of infection to the mucosal nasopharyngeal tissues in the case of MCL, and other skin areas in the case of DCL.

In some cases, parasites bear a cytoplasmic double stranded RNA (dsRNA) virus termed *Leishmania* RNA virus (LRV). There are two known subgenera of LRVs. LRV1 has been found in New World species such as *Lgy* (Tarr et al., 1988) and *Lbr* (Zangger et al., 2013), while another species of LRV, termed LRV2 has been found in Old World *L. major* (Scheffter et al., 1994), *L. aethiopica* (Zangger et al., 2014) and *L. infantum* (Hajjaran et al., 2016). The difference between LRV1 and LRV2 lies within the genetic organization and replication mechanisms (Lee et al., 1996; Se et al., 2005; Fujimura and Esteban, 2011; Kostygov et al., 2021).

Although the existence of such endoprotozoan viruses of the *Totiviridae* family have been discovered and described almost 30 years ago (Tarr et al., 1988; Stuart et al., 1992; Weeks et al., 1992), their role in disease progression remained elusive for years. It is now known that upon its release from dying parasites or possibly due to exosomal transfer (Atayde et al., 2019; Olivier and Zamboni, 2020), this viral endosymbiont is recognized by endosomal TLR3, leading to increased production of pro-inflammatory cytokines and chemokines. Paradoxically, this TLR3-mediated innate response exacerbates the disease as mice infected with LRV1-bearing *Lgy* (*Lgy*LRV1+) develop higher lesions and display more parasite burden at the peak of infection when compared to their LRV1-negative counterparts (*Lgy*LRV1-) (Ives et al., 2011). In part this phenomenon is explained by the activation of Akt-dependent pro-survival mechanisms within the host macrophage. Prolonged survival of infected cells promotes parasite persistence, therefore contributing to the exacerbated phenotype (Eren et al., 2016). We recently showed that LRV1-dependent dissemination and formation of metastatic lesions is linked to low levels of IFN-γ and is mediated by IL-17A (Hartley et al., 2016). Clinical significance of LRV1 presence within *Leishmania* parasites relies on the selection of correct treatment schemes. We and others recently described that patients, diagnosed with MCL and DCL, caused by *Lgy*LRV1+ or *Lbr*LRV1+, require prolonged therapy and are more prone to relapse upon treatment (Adaui et al., 2015; Bourreau et al., 2015). We further identified that type I IFNs, induced downstream of TLR3 signaling, either by LRV1, or co-infecting viruses, are the key molecules that contribute directly to the exacerbated disease phenotype (Rossi et al., 2017).

Being an extremely successful pathogen, *Leishmania* has developed various strategies to evade the host immune response. These include, facilitating its own capture by the host cells and resisting the internal hostile environment together with subverting various signaling pathways and exploiting immune mechanisms in order to facilitate its own survival and progression, thus making *Leishmania* one of the most insidious pathogens (Gupta et al., 2013; Rossi and Fasel, 2018). Some examples describe various strains of *Leishmania* promoting host-cell A20 induction and therefore evading inflammatory reactions as well as suppressing inflammasome activation (Srivastav et al., 2012; Gupta et al., 2017; Hartley et al., 2018).

In the case of *Lgy*LRV1+ infection, viral dsRNA serves as an additional potent immunogen, whose recognition triggers a plethora of signaling cascades. Herein we described how *Lgy*LRV1+ infection initiates a parasiticidal signaling pathway. Consequently, we showed how parasites mitigated these effects and reported an observation implying that *Lgy*LRV1+ may exploit these anti-*Leishmania* mechanisms to facilitate its dissemination.

## Materials and methods

### Ethics statement

All animal protocols were approved by the Swiss Federal Veterinary Office (SFVO), under the authorization licenses 2113 and 3551. Animal experimentation procedures were conducted with strict compliance with the ethical guidelines set out by the SFVO and under inspection by the Department of Security and Environment of the State of Vaud, Switzerland.

### Mice

Four- to five-week-old Wild-type mice (WT) (C57BL/6JOlaHsd) were purchased from Envigo (Netherlands), *Tlr3^-/-^* mice (B6;129S1-Tlr3 tm1Flv/J) were obtained from Prof. S. Akira (Osaka University, Japan) *via* P. Launois (WHO-IRTC, Lausanne, Switzerland), type I IFN receptor-deficient (*Ifnar^-/^* ^-^) mice (B6.129S2-Ifnar1 tm1Agt/Mmjax) were obtained from M.Aguet, Swiss Institute of Experimental Cancer Research (Epalinges, Switzerland), *A20fl/flLySMCre/wt* mice (*A20FL/LysMCre* (Tnfaip3tm1.1Gvl/Lyz2tm1(cre)Ifo)) were obtained from Prof. M. Pasparakis, University of Cologne (Germany), *Ifn-γ^-/-^* (B6.129S7-Ifng tm1Ts/J)and *Inos^-/-^* (B6.129P2-Nos2 tm1Lau/J) mice were purchased from The Jackson Laboratory. *Ifn-γ^-/-^Inos^-/-^* DKO mice were produced by intercrossing *Ifn-γ^-/-^* and *Inos^-/-^* mice. Mice were kept and bred in Green Line cages (Tecniplast) at the animal facility of the Center of Immunity and Immunology, Lausanne (CIIL, Switzerland) in a specific pathogen-free environment. Experiments were performed at a biosafety level 2 (BSL-2) animal facility at the CIIL after one week of acclimatization, in disposable cages (Innovive). Cages were enriched with one igloo, two carboard tubes, one wood stick, and tissues. Males and females were used. In both husbandries, food (SAFE or KLIBA NAFAG) and water were provided ad libitum. Light cycle was maintained at 13 hours light and 11 hours darkness, temperature was set at 21°C ± 2 and humidity was kept at 55% ± 10. *Ifn-γ^-/-^Inos^-/-^* DKO and *A20fl/flLySMCre/wt* mice were genotyped by PCR on tissue-isolated genomic DNA using the 124 KAPA Mouse Genotyping Kit (KAPA Biosystems, USA) using primers shown in **Supplementary Table 1**.

### Parasites

*Lgy*LRV1+ and *Lgy*LRV1- parasites were used in this study. They were obtained following drug treatment of the LRV1+ strain of *Lgy* M4147 containing a firefly luciferase (ffLUC) gene integrated stably into the small subunit gene of the ribosomal RNA locus (LgM4147/SSU : IR2SATLUCb LRV1+) (Kuhlmann et al., 2017). Parasites were cultured at 26°C in a Schneider’s medium (SigmaAldrich) supplemented with 10% FCS, 1% penicillin/streptomycin (P/S), 1% HEPES (Sigma-Aldrich), 0.6 μg/mL of Biopterin (SigmaAldrich) and 5 μg/mL of Haemin (Sigma-Aldrich).

### Macrophage extraction and treatment

Bone marrow derived macrophages (BMDMs) were obtained as follows. Bone marrow was extracted from tibias and femurs of indicated mice. Macrophages were cultured at 37°C and 5% CO2 in 10 mL of Dulbecco’s modified Eagle medium (DMEM, Gibco) supplemented with 10% FCS, 1% P/S, 1% HEPES (Sigma-Aldrich) and 50 ng/mL murine recombinant M-CSF (Immunotools). After 3 d, half of the initial volume of fresh DMEM was added to the culture. 3 d later, BMDMs were seeded on culture plates and incubated overnight. BMDMs were infected with stationary-phase *Lgy*LRV1+ or *Lgy*LRV1- at a multiplicity of infection (MOI) of 10 parasites per macrophage unless stated otherwise. BMDMs were also treated with poly (I:C) (*In vivo*gen) at 2μg/ml unless indicated otherwise, FSL-1 (*In vivo*gen) at 10ng/mL, LPS (*In vivo*gen) at 1μM, IFN-γ (BD Biosciences) at 0.1 U/μl or IFN-β (CellScience) at 500 U/ml.

### Mice infection

Stationary phase parasites were injected into the hind footpads of mice at a concentration of 3 × 10^6^ parasites per footpad in 50 μL of sterile PBS. Footpad thickness was measured weekly post infection (p.i.) using a Vernier caliper. *In vivo* parasites were quantified weekly using Bruker Xtreme II as previously described (Reverte and Fasel, 2019). *Ifn-γ^-/-^Inos^-/-^* DKO mice, *Ifn-γ^-/-^ and Inos^-/-^* mice in experiments **Figures 4C, D**, **6** received Dafalgan (1g/L) diluted in drinking water from week 4.5 p.i. until the end of the experiments*. Inos^-/-^* mice in experiments **Figures 4A, B** were sacrificed at week 6 p.i. for ethical reasons.

### Western blot

Cells after 6, 12- or 24h post-treatment were lysed with. 1.5x Laemmli’s Sample Buffer. Cell lysates were size-fractionated by SDS-PAGE and wet-transferred to a nitrocellulose membrane. The membranes were blocked with 5% milk in Tris buffered saline with 0.1% tween-20 (TBST), and were incubated with primary antibody and with appropriate secondary antibody conjugated to HRP. The membranes were washed with TBST in between incubations. The immunoblot was revealed by the enhanced chemiluminescence Western blotting detection, (GE Healthcare, UK) or with the Fusion Solo S system (Vilber Lourmat). The films (Amersham Hyperfilm, GE Healthcare or SuperRX, Fuji, Japan) were developed using a radiograph (SRX-101a, Konica Minolta). Antibodies used for Western blots are shown in **Supplementary Table 2**.

### mRNA quantification using quantitative real-time PCR

Total RNA was collected by RNAzol RT (Mrcgene). RNA was reverse-transcribed using the PrimeScript RT Reagent Kit with gDNA Eraser (Takara; Clontech Inc., USA). RT-PCR was performed on complementary DNA (cDNA) using primers described in **Supplementary Table 1**. Data were acquired using LightCycler 480 (Roche) and analyzed with the 2^-ΔΔCt^ method. Data were normalized to *L32* expression and samples were calibrated to the expression of the gene of interest.

### RNA sequencing of BMDMs and bioinformatics analysis

BMDMs from WT mice were infected with either strain of *Lgy* parasites or left untreated for 8 hours. RNA was extracted using a RNeasy Kit (Qiagen) following manufacturer’s instructions. RNA quality and concentrations were determined by Fragment Analyzer and Ribogreen QubIT quantification, respectively, and libraries for sequencing were then prepared at the Lausanne Genomic Technologies Facility (GTF). Statistical analysis was performed for genes independently in R (R version 3.5.2). Differential expression was computed with limma (Ritchie et al., 2015) by fitting data to a linear model.

### Cytokine measurements

The concentrations of TNF-α (Invitrogen 88-7324-88), IL-6 (Invitrogen 88-7064-88) in collected supernatants from treated macrophages were determined using enzyme-linked immuno-sorbent assay (ELISA) following the manufacturer’s instructions. The plates (Nunc-Immuno) were read on a Synergy HT Multi-Mode Plate Reader (Biotek Instruments). Wavelength correction and background signals were subtracted from the absorbance values.

### Measurements of cytokine secretion in draining LNs

At indicated time points, lymphocytes were isolated from the popliteal lymph nodes (LNs) draining the primary lesions. Cells (5x10^6^/ml) were stimulated with 10 min UV-irradiated *Lgy*LRV1+ or *Lgy*LRV1- promastigotes (1x10^6^/ml) in complete DMEM medium (Gibco), supplemented with 10% heat-inactivated fetal bovine serum (FBS), 1% penicillin/streptomycin and 1% HEPES (Sigma-Aldrich). After 72 hours, IL-17A cytokines were quantified in cell-free culture supernatants by ELISA (Invitrogen) following the manufacturer’s protocol.

### Measurement of nitric oxide

Nitric oxide concentrations in culture medium were measured with the Griess Reagent System (Promega). Samples (100 µl) were added to the microtiter plates, followed by the addition of 50 µl of 1% sulfanilamide and 50 µl of 0.1% naphthylethylenediamine in 2.5% (vol/vol) H3PO4. After 15 min, absorbance was measured with HT Multi-Mode Plate Reader (Biotek Instruments) at 530 nm and compared with that of a standard curve of NaNO3. Background signal detected in not-infected, not-stimulated condition was subtracted from all experimental conditions.

### High-content microscopy

BMDMs were counted with Vi-Cell (Beckman Coulter), then plated on 96-well tissue-culture-treated clear-bottom plates (Falcon Cat No353219 or Ibidi Cat. No 89626). After overnight incubation, cells were infected with stationary phase *Lgy*LRV1+ or *Lgy*LRV1- for 48 h. Cells were fixed with freshly made 4% PFA in PBS (pH 7.4), stained with DAPI (Molecular Probes) and Alexa 488-Phalloidin (Molecular Probes) then washed with PBS using a Biotek MultioFlo FX plate washer. Forty-nine images (737 square, 0.12 mm2) were acquired from each well using ImageXpress Micro XLS. Cell and parasite numbers were counted using an automated software program (MetaXpress) as previously described (Eren et al., 2018).

## Results

### *Lgy*LRV1+ induced low level expression of iNOS in murine BMDMs with no impact on parasite burden *in vitro*

**Figure 1:**
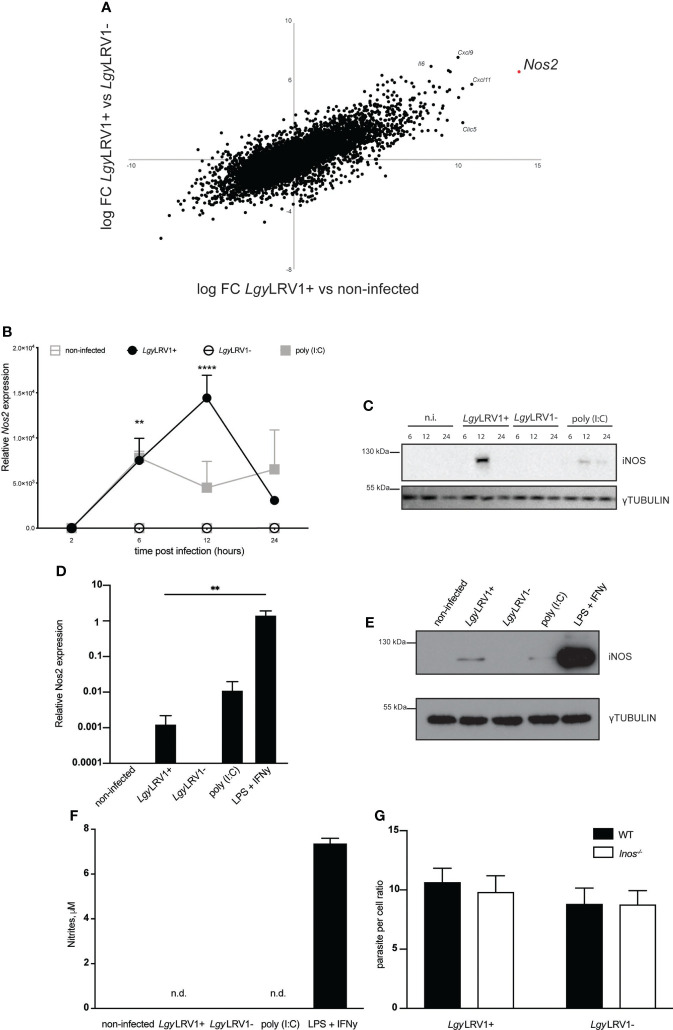
*Lgy*LRV1+ infection induces iNOS expression in murine BMDM. **(A)** WT BMDMs were infected with *Lgy*LRV1+, *Lgy*LRV1- (MOI 5) or left untreated for 8h. Total RNA were extracted and full RNAseq analysis was performed. Scatter-plot shows all present genes comparing *Lgy*LRV1+ vs non-infected (x-axis) and *Lgy*LRV1+ vs *Lgy*LRV1- (y-axis), (n = 4). **(B)** WT BMDMs were infected with *Lgy*LRV1+, *Lgy*LRV1- (MOI 10), stimulated with poly (I:C) (2 μg/ml), or left untreated, for 2, 6, 12 or 24h. RT-PCR was used to measure *L32*-normalized *Nos2* expression. **(C)** WT BMDMs were infected, or treated as in **(A)**. Total cell lysates were analyzed by western blot for iNOS and γ-tubulin expression as loading control at indicated time points. **(D)** WT BMDMs were infected, or treated as in **(A)** for 12h. LPS (1μM) and IFN-γ (0.1 U/μl) stimulation were used as controls. RT-PCR was used to measure *L32*-normalized *Nos2* expression. **(E)** WT BMDMs were infected, or treated as in **(C)**. Total cell lysates were analyzed by western blot for iNOS and γ-tubulin expression as loading control. **(F)** WT BMDMs were infected, or treated as in **(C)**. Upon 48h supernatants were collected and levels of nitrites were measured using Griess assay. **(G)** WT or *Inos^-/-^* BMDMs were infected with *Lgy*LRV1+, or *Lgy*LRV1- (MOI 10) for 48h. Levels of parasitaemia were analysed using high content microscopy. Data show mean ± SD from a representative of three independent experiments **(A, C, E and F)**. Representative blots are shown from at least three independent experiments in **(C, E)** n.i. – non-infected. n.d. - not detected. Data were analyzed using unpaired Student’s t test. Not significant (NS), **p < 0.01, and ****p < 0.0001.

In order to investigate the impact of *Lgy*LRV1+ and *Lgy*LRV1- infection on cellular global transcriptome states, murine bone-marrow derived macrophages (BMDMs) were incubated with *Lgy*LRV1+ and *Lgy*LRV1- for 8h at multiplicity of infection (MOI) =5. A global RNAseq analysis was then performed and the genes expression profiles of *Lgy*LRV1+ infected BMDMs were compared to their *Lgy*LRV1- infected counterparts and to non-infected condition. Several pro-inflammatory genes such as *Il6*, *Cxcl9, Clic5* and *Cxcl11* were strongly upregulated in *Lgy*LRV1+ condition. Strikingly, *Nos2* was the most upregulated gene in case of *Lgy*LRV1+ vs non-infected condition and one of the top upregulated when compared to *Lgy*LRV1- (**Figure 1A**). To verify this result, WT murine bone-marrow derived macrophages (BMDMs) were infected with *Lgy*LRV1+ and *Lgy*LRV1- at a multiplicity of infection (MOI) =10, left untreated or stimulated with poly (I:C). Upon indicated time points total mRNAs were isolated and expression of *Nos2* was measured. Infection of BMDMs with *Lgy*LRV1+ but not with *Lgy*LRV1- led to *Nos2* induction peaking at 12h p.i. The Poly (I:C) control also led to *Nos2* expression (**Figure 1B**). Consequent Western blot analysis revealed expression of the encoded iNOS protein in the aforementioned conditions (**Figure 1C**). Interestingly, despite observable expression of iNOS on the mRNA and protein level (**Figures 1B, C**), conducted Griess assay revealed no nitrites even after prolonged incubation for 48h. At the same time control stimulation with LPS and IFN-γ, which are known iNOS provoking stimuli (Chan and Riches, 2001), led to high levels of iNOS produced at the mRNA (**Figure 1D**) and protein level (**Figure 1E**) and observable levels of nitrites in the supernatants (**Figure 1F**). This indicated that *Lgy*LRV1+ infection or poly (I:C) stimulation led to weak iNOS induction and likely very low levels of nitrites that are not detectable by the Griess assay as the observed values do not exceed the background level detected in the case of the non-infected non-stimulated condition even after prolonged incubation of 48h. Moreover, previously established high content analysis (Eren et al., 2018) revealed no difference in parasite burden between *Lgy*LRV1+ and *Lgy*LRV1- infection of WT as well as iNOS-deficient BMDMs (**Figures 1G**; **Supplementary Figure 1**). These data indicated that in our experimental conditions *Lgy*LRV1+ infection of BMDMs led to iNOS induction with no detectable NO and consequently no impact on parasite burden *in vitro*.

### *Lgy*LRV1+ induced NF-κB signaling appeared to be insufficient to trigger potent iNOS expression

**Figure 2:**
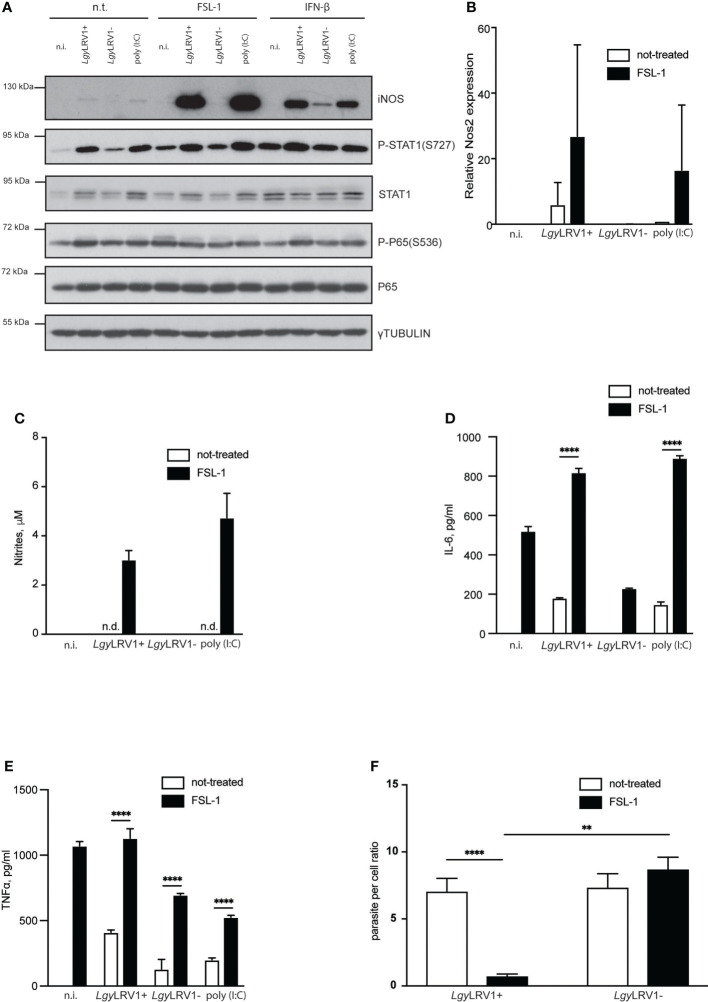
External NF-κB activation boosts *Lgy*LRV1+ triggered iNOS induction and rescues BMDMs from infection. **(A)** WT BMDMs were infected with *Lgy*LRV1+ or *Lgy*LRV1- (MOI 10), left untreated, or stimulated with poly (I:C) (2 μg/ml) for 12h. Additionally some conditions were treated with FSL-1 (10 ng/ml), or IFN-β (500 U/ml), or left untreated. Total cell lysates were analyzed by western blot for iNOS, P-STAT1(S727), STAT1, P65, P-P65(S536) and γ-tubulin expression. **(B)** WT BMDMs were infected as in **(A)**. RT-PCR was used to measure *L32*-normilized *Nos2* expression. **(C–F)** - WT BMDMs were infected as in **(A)** for 48h. Levels of parasitemia were measured by high content microscopy **(F)**. SNs were collected and levels of nitrites **(C)**, IL-6 **(D)** and TNF-α **(E)** were analyzed by Griess assay and ELISA respectively. Data show mean ± SEM **(F)**, or SD **(B–E)** from a representative of three independent experiments. Representative blot is shown from at least three independent experiments in **(A)**. Data were analyzed using unpaired Student’s t test in F or Sidak’s multiple comparisons **(D, E)**. Not significant (NS), **p < 0.01, and ****p < 0.0001. n.i. – non-infected. n.t. – non-treated. n.d. - not detected.

Activation of the transcription factor NF-κB and signal transducer and activator of transcription 1 (STAT-1), and consequent activation of the iNOS promoter are essential steps for iNOS induction (Pautz et al., 2010). We therefore tested whether *Lgy*LRV1+ infection led to phosphorylation of STAT-1 at the S727 residue together with phosphorylation of P65 at S536. Interestingly, both of the aforementioned transcriptional factors were activated upon *Lgy*LRV1+ infection, or Poly (I:C) treatment (**Figure 2A**), indicating that the transcriptional system is functional. We then hypothesized that low expression of iNOS upon *Lgy*LRV1+ infection was due to regulated activation of either one or both required pathways. To test this hypothesis, we infected BMDMs with either strain of *Lgy* and simultaneously provided additional stimuli for the NF-κB or STAT-1 pathways, respectively. To activate STAT-1, we used recombinant interferon beta (IFN-β). FSL-1, a known TLR-2/6 agonist was used in order to provide potent NF-κB stimulation (Okusawa et al., 2004). Strikingly, massive levels of iNOS were observed in the case of a combination of FSL-1 and *Lgy*LRV1+, or FSL-1 and poly (I:C), but not in BMDMs that were treated with FSL-1 and infected with the *Lgy*LRV1- counterparts, or that remained untreated (**Figure 2A**). In contrast, addition of IFN-β of the type I IFN family led to elevated levels of iNOS not only in the case of the *Lgy*LRV1+ and poly (I:C) control, but also in the case of *Lgy*LRV1- infection and not in uninfected cells (**Figure 2A**). We further observed that addition of FSL-1 to *Lgy*LRV1+ infected BMDMs led to the massive increase of *Nos2* transcription (**Figure 2B**), and to detectable levels of nitrites in the culture medium (**Figure 2C**). As expected, stimulation with FSL-1 resulted in activation of NF-kB as indicated by phosphorylation of P65(S536) and increased production of IL-6 and TNF-α in all the conditions (**Figures 2A, D, E**). Moreover, enhanced production of iNOS and nitrites by FSL-1 led to a significant decrease in intracellular infection when BMDM were infected with *Lgy*LRV1+, but not when infected with *Lgy*LRV1- (**Figure 2F**). Various other synthetic TLR-2 agonists had similar effects on nitrite production when added to *Lgy*LRV1+ infected BMDMs (**Supplementary Figure 2A**). Impact on parasite per cell ratio was also assessed for selected ligand and revealed similar results (**Supplementary Figure 2B**). Taken together these data suggested that *Lgy*LRV1+ infection of murine BMDMs resulted in activation of the STAT-1 and NF-kB pathways where the latter is controlled or insufficient to produce large amounts of NO to control infection. This can be bypassed by providing additional NF-kB activating stimuli to provoke robust iNOS induction, providing evidence that in *Lgy*LRV1+ infection, iNOS production is functional but strictly controlled.

### *Lgy*LRV1+ infection induced iNOS expression in murine BMDMs *via* type I IFN signaling

**Figure 3:**
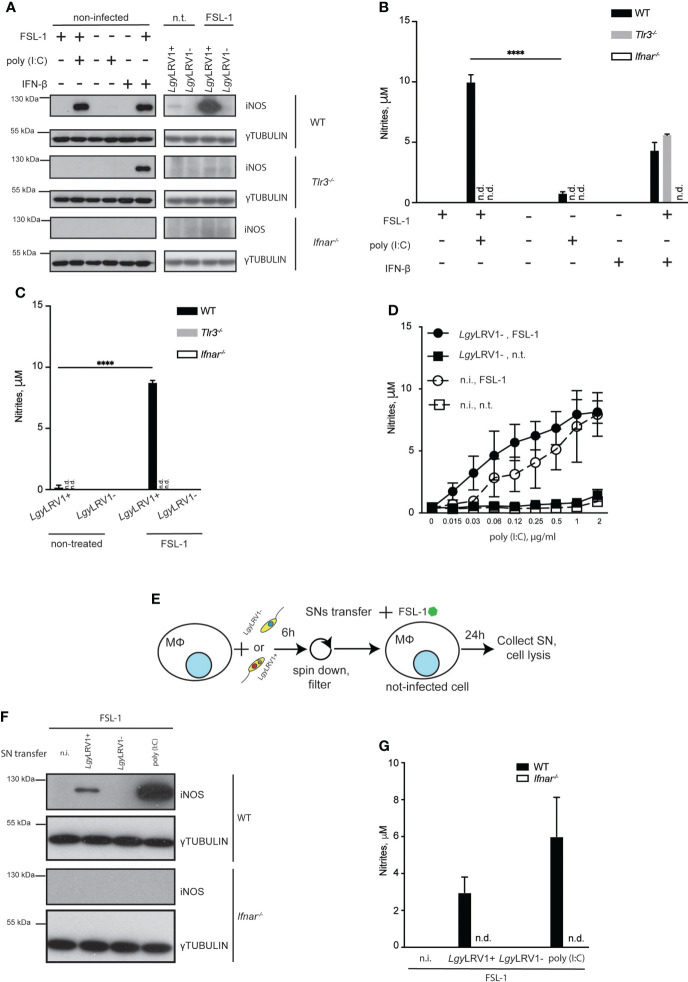
*Lgy*LRV1+ triggered type I IFNs are necessary but not sufficient in order to induce iNOS in an auto- and paracrine manner. **(A)** WT, *Tlr3^-/-^* or *Ifnar^-/-^* BMDMs were infected with *Lgy*LRV1+ or *Lgy*LRV1- (MOI 10), stimulated with poly (I:C) (2 μg/ml), FSL-1 (10 ng/ml), IFN-β (500 U/ml), alone or in combination, or left untreated for 12h. Total cell lysates were analyzed by western blot for iNOS and γ-tubulin expression. **(B, C)** WT, *Tlr3^-/-^* or *Ifnar^-/-^* BMDMs were treated as in **(A)** for 48h. SNs were collected and levels of nitrites were measured using Griess assay. **(D)** WT BMDMs were infected with *Lgy*LRV1- (MOI 10), treated with FSL-1 (10 ng/ml) alone or in combination. Except one, all conditions were treated with poly (I:C), with one of eight two-fold serial dilution concentrations ranging from 2 to 0.015 μg/ml. Upon 48h SNs were collected and levels of nitrites were measured using Griess assay. **(E)** scheme depicting experiments performed in **(F, G)**. **(F, G)** WT BMDMs were infected with *Lgy*LRV1+ or *Lgy*LRV1- (MOI 10), stimulated with poly (I:C) (2 μg/ml), or left untreated. Upon 6h, SNs were collected, filtered with 22 um filter, supplemented with FSL-1 (10 ng/ml) and added to the fresh set of untreated WT and *Ifnar^-/-^* BMDMs. Upon 24h, SNs from treated BMDMs were collected and levels of nitrites were measured using Griess assay **(G)**. Total cell lysates were analysed by western blot for iNOS and γ-tubulin expression **(F)**. Data show mean ± SD from a representative of three independent experiments **(B, C, F)**. Representative blots are shown from at least three independent experiments in **(A, F)**. the following: ****p < 0.0001, n.i. – non-infected. n.t. – non-treated. n.d. - not detected.

We previously reported that *Lgy*LRV1+ infection leads to production of type I IFNs (Ives et al., 2011). iNOS is known to be among the molecules produced in response to type I IFN stimulation (Mattner et al., 2004). Thus, we decided to evaluate if LRV1-induced iNOS was mediated by a second wave of signals arising from type I IFN binding to its receptor (IFNAR). Indeed, macrophages, deficient for IFNAR failed to produce iNOS while cells lacking TLR3 were able to express this enzyme after combined stimulation with recombinant IFN-β and FSL-1 (**Figure 3A**). Consistent with this result, no nitrites were detected in the culture medium of *Ifnar^-/-^* cells, while IFN-β combined with FSL-1 treatment led to nitrite production in *Tlr3^-/-^* cells and WT cells infected with *Lgy*LRV1+ and treated with FSL-1. The same was observed in case of cells that were treated with poly (I:C), or IFN-β in combination with FSL-1, but not alone (**Figures 3B, C**). We also verified that *Lgy*LRV1+ triggered iNOS and nitrite production relied on the presence of LRV1 in parasites and on an LRV1-TLR3-IFNAR signaling axis, and did not depend solely on parasites. WT BMDMs were infected with *Lgy*LRV1- parasites and treated with FSL-1. Simultaneously various concentrations of poly (I:C) were added. Levels of nitrites were detected in a dose-dependent manner in conditions where cells were also treated with FSL-1 (**Figure 3D**). We then hypothesized that the secreted type I IFNs could act on the neighboring non-infected cells and together with external NF-kB activating stimuli could lead to iNOS induction and production of NO. To test this hypothesis, we collected supernatants (SNs) from *Lgy*LRV1+ infected WT BMDMs, supplemented them with FSL-1 and added them to naive WT, or *Ifnar^-/-^* BMDMs (**Figure 3E**). As expected iNOS production and nitrites were observed only in WT cells that were treated with SNs of cells infected with *Lgy*LRV1+, or treated with poly(I:C), and not in *Ifnar^-/-^* cells, or WT cells that were treated with SNs obtained from untreated cells, or from BMDMs infected with *Lgy*LRV1- (**Figures 3F, G**). These data indicated that *Lgy*LRV1+ infection led to iNOS induction *via* the LRV1-TLR3-IFNAR pathway in an auto- and a paracrine manner. Importantly, actions of type I IFNs were indispensable but insufficient as NF-kB stimulation was required to provoke full iNOS induction.

### LRV1-TLR3-IFNAR mediated iNOS did not control *Lgy*LRV1+ infection *in vivo*

**Figure 4:**
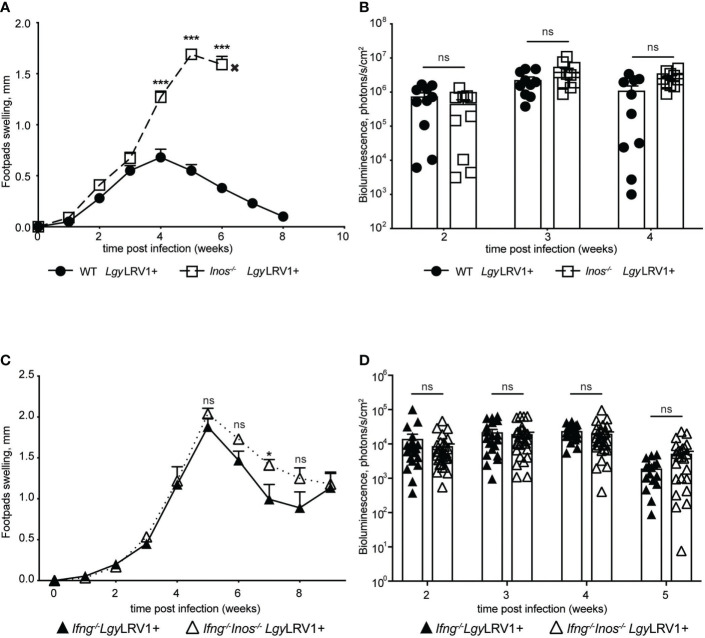
Type I IFNs mediated iNOS does not control *Lgy*LRV1+ infection *in vivo*. Hind footpads of WT, *Inos^-/-^*, *Ifng^-/-^* and *Ifng^-/-^Inos^-/-^* DKO mice were infected with 3 × 10^6^ *Lgy*LRV1+. **(A, C)** – graphs display weekly measurements of footpad swelling. **(B, D)** Parasite burden was determined after infection by bioluminescence imaging. Data show mean ± SEM from representative experiments (n = 4–5 mice) of at least three independent experiments. Each data point on **(B, D)** represents one footpad. Statistical significance is calculated using two-way ANOVA analysis with Bonferonni’s test **(A)**. Not significant (NS), *p < 0.05 and ***p < 0.001.

To further understand the impact of LRV1-TLR3-IFNAR induced iNOS on the course of the disease *in vivo*, we infected iNOS-deficient mice and their WT counterparts with *Lgy*LRV1+ parasites. Interestingly, no difference in footpad swelling, or parasite burden, was observed in the first three to four weeks of infection (**Figures 4A, B**; **Supplementary Figure 3A**) As expected iNOS-deficient mice showed increased levels of footpad swelling and parasitemia around week 4 post infection. After this point WT mice showed decrease in footpad swelling and infection was resolved around week 8 p.i. This can be explained by actions of the Th1 mediated adaptive response that leads to production of IFN-γ and consequent control of infection *via* iNOS (Sacks and Noben-Trauth, 2002). It was, however, not possible to fully determine the moment when CD4+ Th1 cells began to produce IFN-γ leading to iNOS production. Moreover, NK cells are a major source of IFN-γ during innate phase long before the development of an adaptive response (Biron and Brossay, 2001). Thus, in order to investigate the exact impact of iNOS induced by *Lgy*LRV1+ and not induced by other stimuli, we needed to exclude the role of IFN-γ, as the iNOS inducer. With this in mind we generated *Ifn-γ^-/-^Inos^-/-^* double knock out (DKO) mice. We then infected DKO and *Ifn-γ^-/-^* single deficient mice with *Lgy*LRV1+ parasites. In IFN-γ-deficient mice, the LRV1-TLR3-IFNAR axis should remain the predominant signaling axis to induce iNOS. Strikingly both genotypes responded to infection in a similar pattern with almost no significant difference in footpad swelling or parasite burden (**Figures 4C, D**; **Supplementary Figure 3B**). These findings showed that iNOS triggered by *Lgy*LRV1+ did not control infection *in vivo*.

### *Leishmania guyanensis* suppressed type I IFN activated iNOS expression by inducing A20 and minimizing NF-kB signaling

**Figure 5:**
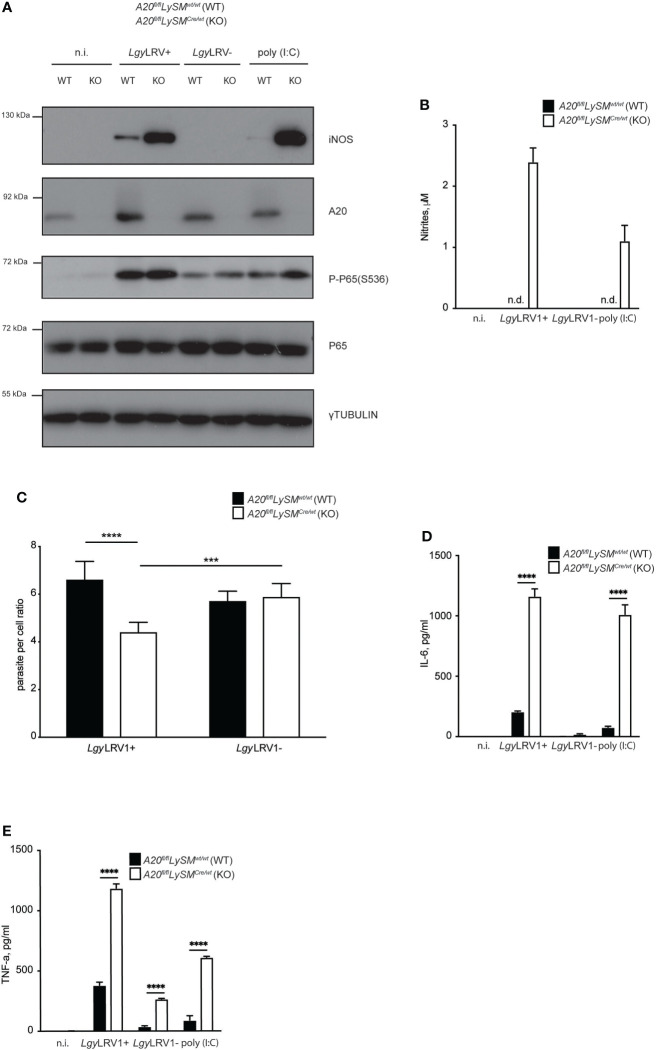
*Lgy* infection minimizes iNOS expression by suppressing NF-κB *via* A20 induction. **(A)** WT or *A20^fl/fl^LySM^Cre/wt^* (KO) BMDMs were infected with *Lgy*LRV1+, or *Lgy*LRV1- (MOI 10), stimulated with poly (I:C) (2 μg/ml), or left untreated for 12h. Total cell lysates were analysed by western blot for iNOS, A20, P-P65(S536), P65 and γ-tubulin expression. **(B–E)** - WT or (KO) BMDMs were treated as in **(A)** for 48h. Levels of parasitemia were measured by high content microscopy **(C)**. SNs were collected and levels of nitrites **(B)**, IL-6 **(D)** and TNF-α **(E)** were analyzed by Griess assay and ELISA, respectively. Data show mean ± SD from a representative of three independent experiments **(B–E)**. Representative blot is shown from at least three independent experiments in **(A)**. Statistical significance is calculated using unpaired Student’s t test in **(C)** and Tukey’s multiple comparison test in **(D)** and **(E)**. Not significant (NS), ***p < 0.001, ****p < 0.0001. n.i. – non-infected. n.t. – non-treated. n.d. - not detected.

We recently showed that both *Lgy*LRV1+ and *Lgy*LRV1- strains trigger A20 production (Hartley et al., 2018), an early NF-kB induced gene that is known to be a major negative feedback regulator of NF-kB-mediated inflammation (Coornaert et al., 2009; Catrysse et al., 2014). Therefore, we set to investigate the impact of *Lgy*LRV1+ induced A20 on NF-kB suppression and consequent downregulation of iNOS. Full A20 knockout mice rapidly become cachectic and died within a few weeks of age, as a result of uncontrolled inflammation in several organs (Lee et al., 2011). Thus, we used BMDMs obtained from *A20^fl/fl^LysMCre^+/wt^* mice. These animals have a deletion of A20 in the myeloid lineage. *A20^fl/fl^LysMCre^wt/wt^* mice were used as littermate control. In line with reported previously results (Hartley et al., 2018), both strains of *Lgy* induced A20 expression, with *Lgy*LRV1+ showing stronger expression (**Figure 5A**). When infected with either strain of *Lgy*, *A20^fl/fl^LysMCre^+/wt^* cells showed stronger signs of inflammation, including higher levels P-P65(S536) and increased production of IL-6 and TNF-α (**Figures 5A, D, E**). As expected, A20-deficient cells that were infected with *Lgy*LRV1+, but not *Lgy*LRV1- parasites, showed stronger expression of iNOS (**Figure 5A**). Moreover, significant amounts of nitrites were detected in the culture medium as well as a decrease in parasite per cell ratio (**Figures 5B, C**). These data indicated that *Lgy*LRV1+ infection led to A20 activation which in turn limited NF-kB activation, therefore minimizing LRV1-TLR3-IFNAR mediated iNOS induction and parasite death.

### *Ifn-γ^-/-^Inos^-/-^* double deficient mice showed delayed formation of metastatic nodules upon *Lgy*LRV1+ infection

**Figure 6:**
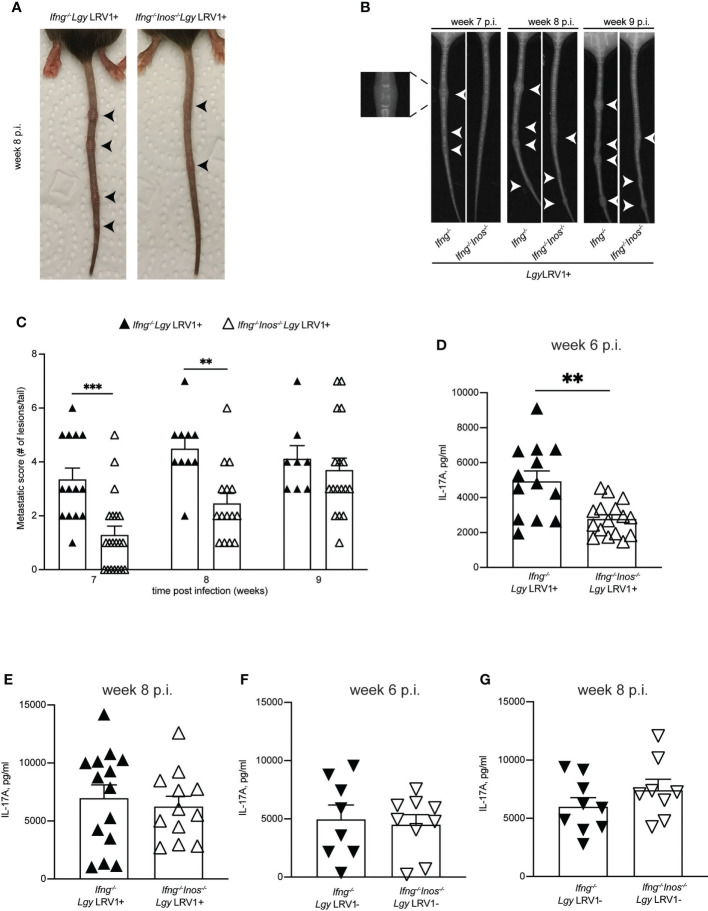
*Ifng^-/-^Inos^-/-^* mice showed delayed metastasis formation compared to *Ifng^-/-^* upon *Lgy*LRV1+ infection. Hind footpads of *Ifng^-/-^* and *Ifng^-/-^Inos^-/-^* DKO mice were infected with 3 × 10^6^ stationary phase promastigotes of *Lgy*LRV1+. **(A)** Photographic images of representative mice showing metastatic lesions on the tail at week 8 post infection. Arrow heads indicate the metastatic nodules. bone destruction. **(B)** X-ray images of representative mice showing metastatic lesions on the tail at indicated time points. Arrow heads and enlarged image indicate bone destruction. **(C)** Number of metastatic lesions per mouse. **(D–G)** *Ifng^-/-^* and *Ifng^-/-^Inos^-/-^* DKO mice were infected into hind foot pads with 3 × 10^6^ stationary phase promastigotes of *Lgy*LRV1+ or *Lgy*LRV1- as indicated. At 6- and 8-weeks post infection, lymphocytes were extracted from popliteal LNs draining the primary lesion and then re-stimulated *ex vivo* with UV-irradiated promastigotes of *Lgy*LRV1+ or *Lgy*LRV1- respectively. Upon 72h, IL-17A secretion was quantified by ELISA in cell-free supernatants. Data show mean ± SEM from three independent experiments with at least 3 mice per group for *Lgy*LRV1+ infection and from two independent experiments with at least 3 mice per group for *Lgy*LRV1- infection. Statistical significance is calculated using 2-way Anova test in **(C)** and Student’s t test in **(D–G)**. Not significant (NS), **p < 0.01, and ***p < 0.001.

As described above *Ifn-γ^-/-^Inos^-/-^* DKO mice showed no difference in footpad swelling or parasite load compared to IFN-γ deficient mice (**Figures 4C, D**). This suggested that LRV1-TLR3-IFNAR triggered iNOS had very little impact on parasite control. Surprisingly, double deficient mice showed fewer metastatic nodules on the tail compared to IFN-γ deficient mice. Nodules appeared less severe (**Figure 6A**), smaller in size and their formation was delayed by at least one week (**Figure 6C**). X-ray imaging also revealed that formation of nodules was accompanied by partial bone destruction and occurred earlier than its visible appearance on the tail (**Figure 6B** enlarged segment). We previously reported that IFN-γ deficient mice produce large amounts of IL-17A upon *Lgy*LRV1+ infection and that this cytokine governs the formation of the metastatic nodules as evidenced by its genetical deletion or chemical inhibition that dramatically reduced the number of metastatic nodules (Hartley et al., 2016). *Ifn-γ^-/-^Inos^-/-^* DKO mice showed decreased production of IL-17A in the draining lymph nodes at week 6 p.i. (**Figure 6D**). No difference was observed at a later time point (week 8 p.i.) (**Figure 6E**) as well as when mice were infected with *Lgy*LRV1- parasites (**Figures 6F, G**). Although not significant, we observed a tendency showing fewer parasites in the tails of *ifn-γ^-/-^inos^-/-^* mice compared to their *ifn-γ^-/-^* counterparts (**Supplementary Figure 4**). These data suggested that NO produced downstream of the LRV1-TLR3-IFNAR-iNOS signaling axis might affect secretion of IL-17A and thus impact formation of secondary metastatic lesions.

## Discussion

Although transmission of most CL-causing *Leishmania* spp is zoonotic by nature (Gramiccia and Gradoni, 2005), generations of man-made impact on environmental setting and urbanization have led to the disease being acquired in various types of human settlements (Reithinger et al., 2019). This led to wider geographical distribution and increased incidence of human cases that were first described as early as 7^th^ century BC (Cox, 2002; Zink et al., 2006). Threatening humanity for thousands of years, *Leishmania* parasites have excelled at survival and propagation. In a long lasting evolutional race they have developed various mechanisms allowing them to evade and subvert defensive mechanisms of the host (Aguirre-Garcia et al., 2018; Rossi and Fasel, 2018). One strategy lies within the cooperative relationship with dsRNA viruses such as LRV1 that are harbored inside the parasite. Innate immune recognition of LRV1 within *Lgy* leads to exacerbation of the disease (Ives et al., 2011), prolonged survival of the host cell and persistence of intracellular parasites (Eren et al., 2016) that in turn facilitates parasite dissemination and metastasis (Hartley et al., 2016). Similarly, infection with exogenous viruses can seriously worsen the course of disease, promote metastasis and relapse of leishmaniasis caused by LRV1-free *Lgy* (Rossi et al., 2017).

An indispensable role of iNOS in immune protection against leishmaniasis in mice has been long known (Wei et al., 1995), and widely reviewed (Bogdan, 2015; Olekhnovitch and Bousso, 2015). Host defense against leishmaniasis primarily relies on the actions of a mounted adaptive immune response. Several weeks upon infection, C57BL/6 mice develop a strong type 1 helper (Th1) CD4+ T cell response. These mice are able to efficiently control infection whereas BALB/c mice develop a type 2 helper (Th2) CD4+ T cell response and fail to recover. These observations provided evidence that immune protection against leishmaniasis is governed by actions of CD4+ T cells (Sacks and Noben-Trauth, 2002). Upon polarization, CD4+ T cells migrate to the lesion where they accumulate (Filipe-Santos et al., 2009). Consequently, they begin secreting large amounts of IFN-γ of the type II IFN family. Sensed by macrophages, this cytokine triggers expression of iNOS which in turn generates high amounts of NO leading to parasite elimination in an auto- and paracrine manner (Liew et al., 1991; Olekhnovitch and Bousso, 2015).

In this study, we revealed that one of the major impacts of LRV1 recognition by TLR3 was up-regulation of iNOS expression. However, LRV1-triggered iNOS failed to control infection *in vitro* and *in vivo*. We further showed that production of iNOS in response to LRV1 required the action of type I IFNs, that have been previously shown to govern the exacerbated disease phenotype (Rossi et al., 2017). We then investigated the strategy employed by the parasites to evade death by type I IFN-mediated iNOS. We showed that *Leishmania* suppressed NF-kB activation, another pathway required for a robust iNOS induction. Finally, we report an observation suggesting that low levels of LRV1-triggered iNOS and, the production of NO that might correlate with parasite propagation and metastasis. Our findings provide the link between disease exacerbating LRV1-elicited type I IFNs and IL-17A mediated metastasis, occurring in the absence, or in the presence of low levels of protective IFN-γ.

Type I IFNs that are mainly associated with innate immune response are also known to be upstream of iNOS induction. Their role in pathogenesis of leishmaniasis is controversial and, most likely, depends on the species of *Leishmania*. In particular, IFN-β has been shown to be protective against *Leishmania major* (*Lm*) of the *Leishmania* subgenus. This effect has been associated with iNOS induction (Diefenbach et al., 1998; Mattner et al., 2004). On the other hand, species of the *Leishmania Viannia* subgenus such as *Lbr* and *Leishmania amazonensis* (*Lam*) benefit from the actions of type I IFNs in an NO-independent manner. In the latter case, type I IFNs led to up-regulation of superoxide dismutase I (SOD1) and consequent reduction of parasiticidal superoxide (Khouri et al., 2009). We recently reported that LRV1-induced type I IFNs contributed to disease progression in the case of *Lgy* infection (Rossi et al., 2017).

The exact mechanism leading to type I IFN-mediated iNOS induction is based on cooperation of two transcriptional pathways and has been well characterized (Farlik et al., 2010; Wienerroither et al., 2015). On the one hand it involves the type I IFN pathway, whereby ligation of IFNAR leads to phosphorylation of STAT1 and STAT2 by the receptor-associated Janus tyrosine kinases (JAK). The tyrosine-phosphorylated STATs form heterodimers and also bind to the interferon regulatory factor 9 (IRF9). This results in the formation of a trimeric complex, known as interferon-stimulated gene factor 3 (ISGF3). ISGF3 alone may be both necessary and sufficient for the transcription of some interferon-stimulated genes (ISGs). However, often and depending on a promoter, it requires input from additional signaling pathways such as NF-kB (Levy and Darnell, 2002). This becomes the second arm of the aforementioned transcriptional cooperation, which is necessary for iNOS induction. NF-kB binds to the gene promoter following induction by PRR signaling, this in turn leads to the recruitment of various proteins involved in the formation of a bigger transcriptional complex. Consequent assembly and binding of ISGF3 leads to the ligature of previously recruited proteins to their binding partners and later recruitment of complexes containing the RNA polymerase II (Pol II) (Farlik et al., 2010; Wienerroither et al., 2015). Our data lied within this mechanism whereby LRV1-induced type I IFNs could be sensed by BMDMs in an autocrine and paracrine manner. However, the NF-kB pathway appeared to be controlled, or insufficient to lead to strong iNOS induction *in vitro*. Our data demonstrated that the above described system was not impaired functionally as additional NF-kB stimulation *via* TLR-2/6 signaling boosted LRV1-mediated iNOS induction (**Figure 2**).

It is evident that the overall impact of the LRV1-TLR3-IFNAR signaling axis benefited disease progression and contributed to the pathological phenotype (Rossi et al., 2017). iNOS can also be induced by activation of TLR4 (Abdul-Cader et al., 2016), or as shown by our data, by combined ligation of TLR2 and TLR3 (**Figures 3A, B**). We previously demonstrated that TLR2 and TLR4 do not play major roles in the innate response to *Lgy* (Ives et al., 2014). This was also supported by another study which showed no protective role of TLR2 in response to *Lbr*, another species of the *Leishmania Viannia* subgenus (Vargas-Inchaustegui et al., 2009). These findings were opposite to what is known in regard to TLR2 function against the *Leishmania* subgenus and *Lm* in particular (Halliday et al., 2016). Observed species-specific differences can be explained by the fact that *Leishmania* parasites express different types, and up to 10-fold less lipophosphoglycan (LPG), a surface molecule, which is known to activate TLR-2 (Muskus et al., 1997; De Assis et al., 2012). Taken together these results lead to the assumption that the symbiotic partnership between *Leishmania* parasites and LRV1 occurred preferentially in those parasite species where the chances to induce parasitotoxic iNOS were minimized and thus allowed beneficial effects of co-habitation that overweighed the detrimental ones.

As described above, BMDM infection with *Lgy*LRV1+ alone led to very weak iNOS production at the protein level and no detectable nitrites in the culture medium that did not have an impact on the level of intracellular infection *in vitro* (**Figures 1E–G**). This observation combined with the *in vivo* data (**Figures 4C, D**) led us to hypothesize that *Lgy*LRV1+ parasites developed a strategy to minimize type I IFN induced iNOS, allowing them to benefit from other effects of type I IFN signaling (Rossi et al., 2017). As we were able to greatly enhance *Lgy*LRV1+ induced iNOS induction by providing additional inflammatory stimuli (**Figures 2A–F**), we assumed that the iNOS-coping strategy of *Lgy*LRV1+ lied within suppression of NF-kB activation. While artificial in our *in vitro* setting, such additional inflammatory stimuli can naturally arise from harmless commensals present in the skin and known to impact inflammatory reactions (Naik et al., 2012; Belkaid and Tamoutounour, 2016; Gimblet et al., 2017) or from sandfly gut microbiota egested together with the parasites (Dey et al., 2018). Such immunologically cross-reactive environment would serve as an additional stimulus for a parasite to evolutionary develop iNOS-mitigation mechanisms.

TNFAIP3, also known as A20, is a ubiquitin-modulating enzyme known to regulate NF-kB activity by removing the K63-linked polyubiquitin chains from RIPK1 and thus interfering with its interaction with IKK-γ (Karin and Greten, 2005), a crucial element in the classical NF-kB pathway. Several studies reported that *Leishmania* infection is able to promote A20 induction and thus control NF-kB activity and subsequently evade iNOS induction (Srivastav et al., 2012; Gupta et al., 2017). We recently showed that both, *Lgy*LRV1+ and *Lgy*LRV1- strains, induce A20 expression (Hartley et al., 2018). Notably, A20 production was more potent in the first hours of infection and was intensified in case of *Lgy*LRV1+. This, together with the fact that A20 expression is itself under the control of NF-kB (Catrysse et al., 2014) suggested two waves of signaling. Firstly, infection with *Lgy*LRV1+ led to production of type I IFN and activation of NF-kB that consequently led to A20 induction. Secondly, A20 suppressed NF-kB activity before the LRV1-triggered type I IFNs were sensed by IFNAR and led to formation of ISGF3 and induction of iNOS. To test this hypothesis we used BMDMs obtained from mice conditionally lacking A20 in myeloid lineage as full A20 knockout mice rapidly become cachectic and died within a few weeks of age, as a result of uncontrolled inflammation in several organs (Lee et al., 2011). We revealed that, indeed, cells lacking A20 expressed higher amounts of pro-inflammatory cytokines, iNOS and NO and were able to resist to *Lgy*LRV1+ but not to *Lgy*LRV1- infection (**Figures 5A–E**). While we could prove this hypothesis *in vitro*, *in vivo* experiments were impossible for ethical reasons as A20 deficient mice spontaneously developed polyarthritis over time (Vande et al., 2014) and any parasitic infection would extremely aggravate this effect. Taken together, we concluded that *Lgy*LRV1+ parasites efficiently evaded LRV1-TLR3-IFNAR-triggered iNOS induction by activating A20 expression and thus suppressing NF-kB which is also required for potent iNOS production. Therefore, we provided, for the first-time evidence of how a symbiotic relationship between the protozoan parasite *Leishmania* and its dsRNA virus, LRV1, can lead to potentially mutually-detrimental effects of this cohabitation and how this mechanism was circumvented.

The exact mechanisms that govern parasite dissemination processes in MCL and DCL complications of the cutaneous form of the disease are largely unknown and the role of LRV1 is still controversial. Host-related immunological and environmental as well as parasite species-specific factors all have been linked to mediation of infectious metastasis, suggesting that this process is multifactorial and depends on a large number of players and their interaction (Hartley et al., 2014). LRV1, nested within *Lgy* and *Lbr* has been shown to be one of such factors and, perhaps, the main one among those associated with the parasite. *In vivo* experiments showed that presence of LRV1 strongly enhances metastatic activity of the parasites in hamsters and mice (Martinez et al., 2000; Ives et al., 2011; Hartley et al., 2016) although the latter were immunocompromised. LRV1 was found in various clinical isolates of *Lgy* and *Lbr* from Neotropical regions and was linked to the metastatic phenotype in patients (Cantanhêde et al., 2015), although this association was not exclusive (de Oliveira Ramos Pereira et al., 2013; Saberi et al., 2019; Valencia et al., 2022). These discrepancies further suggest that host-related factors such as the strength of the immune system seem to play a major role in mediating parasitic metastasis. We recently reported that patients, suffering from chronic forms of leishmaniasis caused by *Lgy* had decreased levels of IFN-γ and high levels of the inflammatory cytokine IL-17A. Moreover, these ratios were strictly dependent on whether parasites contained LRV1 or not (Hartley et al., 2016). Based on this we earlier established a murine model to study metastatic leishmaniasis and observed that absence, or low levels of IFN-γ, was a prerequisite for the metastatic effect whilst presence of LRV1 significantly intensified it. In the absence of IFN-γ, levels of IL-17A in the draining lymph nodes were highly elevated and seemed to govern the development of metastasis as genetic deletion or pharmacological blockade of this cytokine abolished the effect (Hartley et al., 2016). Similarly, involvement of IL-17A in metastatic processes is well documented in cancer research (Coffelt et al., 2015; Song et al., 2020; Zhao et al., 2020).

In our study we wished to determine whether LRV1-TLR3-IFNAR induced iNOS is able to control *Leishmania* infection *in vivo*. As STAT-1 activation, required for *Nos2* transcription, could be induced by either type I or type II IFN signaling, we needed to exclude the latter in order to answer the aforementioned question. Thus, we used IFN-γ-deficient mice, in which the LRV1-TLR3-IFNAR axis should remain the predominant signaling path to induce iNOS. Additionally, we generated *Ifn-γ* ^-/-^ *Inos* ^-/-^ DKO mice that would lack iNOS and have the same genetic background. Both genotypes responded to infection in a similar pattern with no difference in footpad swelling or parasite burden (**Figures 4C, D**) and thus confirming the previously stated conclusion that LRV1-triggered iNOS is insufficient to control the infection due to suppression of NF-kB by parasite-induced A20. However, the difference that we observed was in the formation of metastatic lesions on the tail that should arise in IFN-γ-deficient mice as described above (Hartley et al., 2016). DKO mice developed less severe nodules on the tail and their formation was delayed by 1-2 weeks (**Figures 6A-C**). In line with this delay we observed that DKO mice had decreased levels of metastasis-mediating IL-17A in the draining lymph nodes compared to *Ifn-γ* ^-/-^ deficient mice (**Figure 6D**). This effect was transient as at the later time point no significant difference was observed (**Figure 6E**). This is consistent with the appearance of the metastatic nodules on the tails (**Figure 6C**). Although, we could not observe significant reduction of the parasite load on the secondary sites (tail) (**Supplementary Figure 4**), our findings suggest that LRV1-triggred iNOS and the NO produced by it may have pro-metastatic effects in IFN-γ-deficient mice. This observation correlates well with the role of NO in the field of cancer metastasis. Pro-metastatic effects of NO are specifically attributed to the low concentrations (Tozer et al., 1997; Felley-Bosco, 1998; Cheng et al., 2014), while high concentrations have an opposite, anti-metastatic and tumoricidal effect (Juang et al., 1995; Dong et al., 1994). Our data lies within these concentration-dependent effects as LRV1-TLR3-IFNAR induced iNOS and subsequently produced NO are barely detectable in *in vitro* experiments and their amounts are insufficient to control the infection.

In our experiments we observed that depletion of the *Nos2* gene in addition to I*fn-γ^-/-^* leads to decreased IL-17A production and delayed metastasis. There is little and contradicting evidence of a direct cause-effect association between iNOS and IL-17A. While some studies show the positive correlation between iNOS and stability of Th17 cells as well as production of IL-17A (Obermajer et al., 2013; Zhong et al., 2018), others show that NO negatively regulates these processes (Niedbala et al., 2011). While these discrepancies might also relate to the magnitude of NO, it is important to mention that we were unable to detect the exact T cell population that was responsible for IL-17A production in *Lgy*LRV1+ infection (Hartley et al., 2016). Thus, the exact relation between LRV1-triggred iNOS and IL-17A production in *Lgy*LRV1+ infection of *Ifn-γ-/-* mice is still unclear and requires further investigation.

In conclusion we demonstrated that LRV1-bearing *Leishmania guyanensis* induced A20 to minimize NF-kB activation and evaded death *via* iNOS induced by its viral endosymbiont. Additionally, we report that low amounts of NO induced as a result of the LRV1-TLR3-IFNAR signaling axis might correlate with the amounts of metastasis mediating IL-17A and thus impact parasite metastasis in an immunocompromised setting.

## Data availability statement

The datasets presented in this study can be found in online repositories. The names of the repository/repositories and accession number(s) can be found below: https://www.ncbi.nlm.nih.gov/, GSE201120.

## Ethics statement

The animal study was reviewed and approved by All animal protocols were approved by the Swiss Federal Veterinary Office (SFVO), under the authorization licenses 2113 and 3551. Animal experimentation procedures were conducted with strict compliance with the ethical guidelines set out by the SFVO and under inspection by the Department of Security and Environment of the State of Vaud, Switzerland.

## Author contributions

DK and NF designed research. DK, CD, FP, MR, RM, TS, and SC performed research. RO, L-FL, MP and SB contributed animals, reagents/analytic tools. DK analyzed data. DK and NF wrote the paper. All authors contributed to the article and approved the submitted version.

## Funding

This work is funded by the grants from the Swiss National fund for research (http://www.snf.ch/en/Pages/default.aspx) (No. 310030_173180 and IZRJZ3_164176/1 to NF) and the National Institute of Health (NIH, https://www.nih.gov/) (R01AI-31078 and R01AI-30222-02, SB).

## Acknowledgments

We thank Dr. Dimitri Moreau from the NCCR Geneva Access platform for his help with high-content imaging analysis. We are very grateful to Dr. Stephanie Claudinot and all the veterinary and animal care-taking staff of the University of Lausanne and especially Margaux Baudet for maintaining the highest ethical standards for animal experimentation. We are thankful to Rebecca Balz for technical help with some experiments and her cheerful spirit. We thank Dr. Nathalie Isorce, Dr. Amel Bekkar and Dr. Filipa Teixeira for providing RNAseq data. We also thank Dr. Slavica Masina and for careful reading of the manuscript.

## Conflict of interest

The authors declare that the research was carried out in the absence of any commercial or financial relationships that could result in a potential conflict of interest.

## Publisher’s note

All claims expressed in this article are solely those of the authors and do not necessarily represent those of their affiliated organizations, or those of the publisher, the editors and the reviewers. Any product that may be evaluated in this article, or claim that may be made by its manufacturer, is not guaranteed or endorsed by the publisher.

## Supplementary material

Supplementary Figure 1 Related to **Figure 1G**. WT or *Inos^-/-^* BMDMs show similar levels of parasitemia upon 48h infection with wither strain of *Lgy* (MOI 10). Representative images of HC microscopy. DAPI staining nuclei of the cells and intracellular parasites is shown in green. Phalloidin staining BMDM cytoplasm is shown in red.

Supplementary Figure 2 Related to **Figure 2**. TLR2 stimulation of *Lgy*LRV1+ infected BMDM enhances iNOS induction and rescues BMDMs from infection. **(A)** WT BMDMs were treated with *Lgy*LRV1+ or *Lgy*LRV1- (MOI 10), left untreated or stimulated with poly (I:C) (2 μg/ml). Simultaneously the cells were treated with various TLR2 synthetic agonists at concentrations indicated in **Supplementary Table 3**. Upon 48h, SNs were collected and levels of nitrites were measured using Griess assay. **(B)** WT BMDM were treated with *Lgy*LRV1+, or *Lgy*LRV1- (MOI 10), left untreated or stimulated with poly (I:C) (2 μg/ml). Simultaneously the cells were treated with heat killed Staphylococcus epidermidis (HKSE) (10^7^ cells/ml) for 48h. Levels of parasitemia were measured by high content microscopy. Data are representative from at least three independent experiments. Statistical significance is calculated using Student’s t test. Not significant (NS), ****p < 0.0001.

Supplementary Figure 3 Related to **Figure 4** *Ifng^-/-^* and Ifng^-/-^Inos^-/-^ mice display similar levels of parasitemia upon *Lgy*LRV1+ infection. Hind footpads of (A) WT and *Inos^-/-^* and (B) *Ifng^-/-^* and *Ifng^-/-^Inos^-/-^* DKO mice were infected with 3 × 10^6^ stationary phase promastigotes of *Lgy*LRV1+. Bioluminescence was quantified using Bruker Xtreme II as previously described (Reverte and Fasel, 2019). Representative images of bioluminescence are shown at the selected time point (3 weeks p.i.).

Supplementary Figure 4 Related to **Figure 6**. *Ifng^-/-^Inos^-/-^* mice showed tendency for decreased levels of parasitemia in the tails compared to *Ifng^-/-^* upon *Lgy*LRV1+ infection. Parasite burden at the tails was measured at week 8 p.i. by RT-qPCR, measuring *Kmp11* gene expression. Data is representative from 2 independent experiments with 5 mice per group. Statistical significance is calculated using Student’s t test. Not significant (NS)

Supplementary Table 1 Oligonucleotides used in this study.

Supplementary Table 2 Antibodies used in this study

Supplementary Table 3 TLR agonists concentrations used in **S1A, B Fig**.
