# Supplementary figures and images for: Leishmania guyanensis suppressed inducible nitric oxide synthase provoked by its viral endosymbiont
Source: Front Cell Infect Microbiol. 2022 Aug 12;12:944819. doi: 10.3389/fcimb.2022.944819 (PMC9416488; doi:10.3389/fcimb.2022.944819)

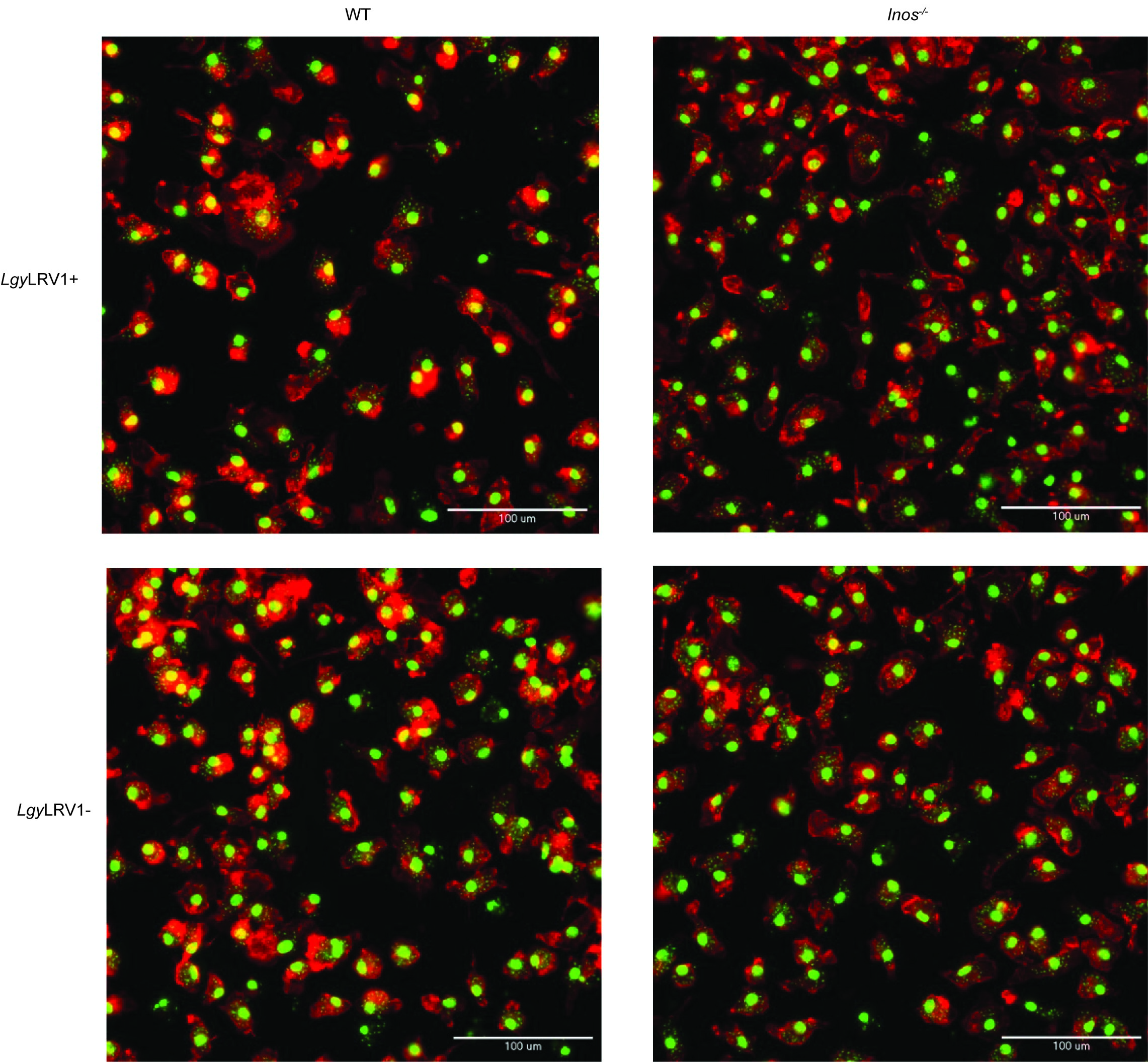

Supplement: Supplementary Figure 1 — Related to Figure 1G . WT or Inos-/- BMDMs show similar levels of parasitemia upon 48h infection with wither strain of Lgy (MOI 10). Representative images of HC microscopy. DAPI staining nuclei of the cells and intracellular parasites is shown in green. Phalloidin staining BMDM cytoplasm is shown in red. [file Image_1.jpeg]

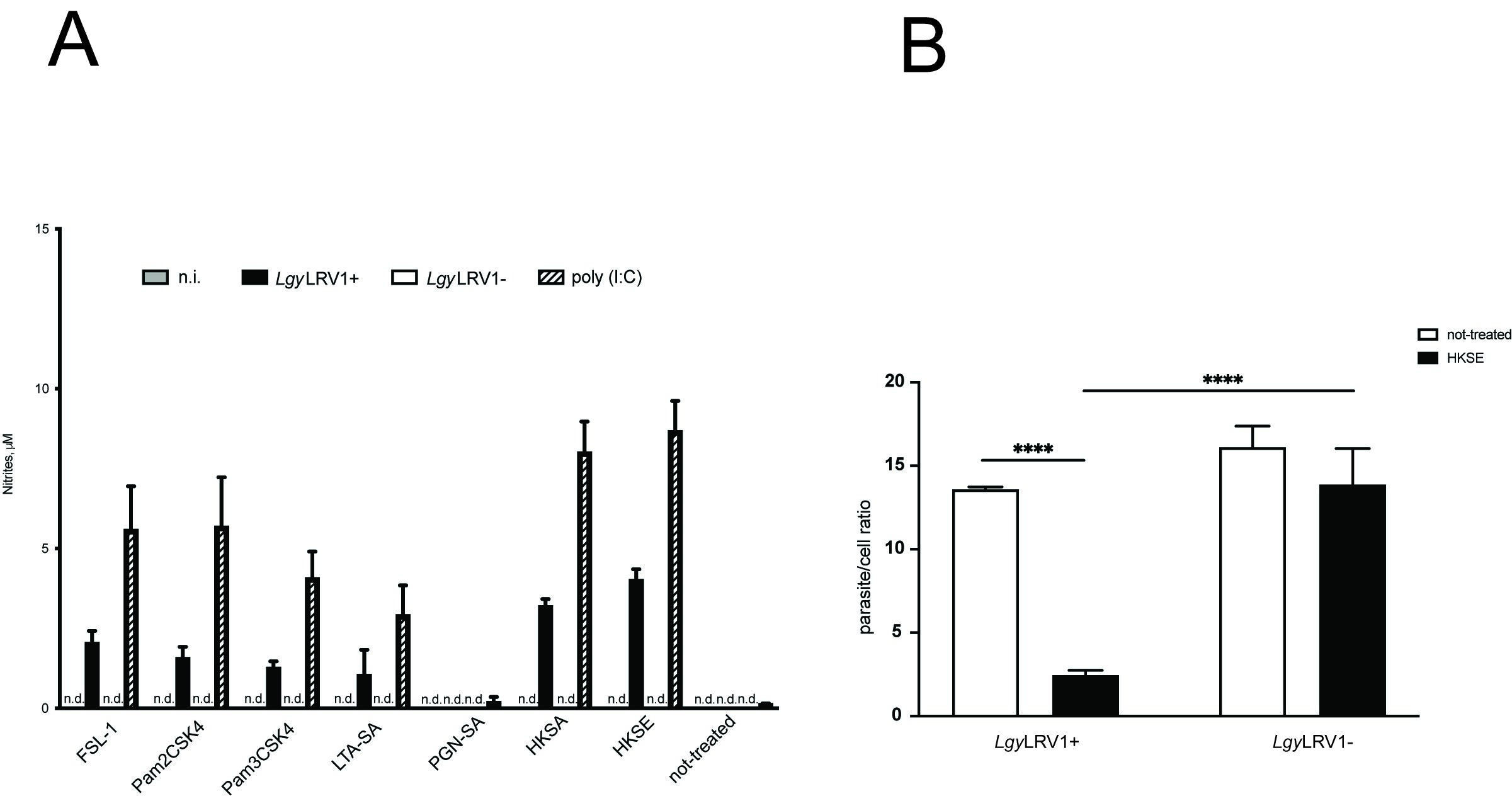

Supplement: Supplementary Figure 2 — Related to Figure 2 . TLR2 stimulation of LgyLRV1+ infected BMDM enhances iNOS induction and rescues BMDMs from infection. (A) WT BMDMs were treated with LgyLRV1+ or LgyLRV1- (MOI 10), left untreated or stimulated with poly (I:C) (2 μg/ml). Simultaneously the cells were treated with various TLR2 synthetic agonists at concentrations indicated in Supplementary Table 3 . Upon 48h, SNs were collected and levels of nitrites were measured using Griess assay. (B) WT BMDM were treated with LgyLRV1+, or LgyLRV1- (MOI 10), left untreated or stimulated with poly (I:C) (2 μg/ml). Simultaneously the cells were treated with heat killed Staphylococcus epidermidis (HKSE) (107 cells/ml) for 48h. Levels of parasitemia were measured by high content microscopy. Data are representative from at least three independent experiments. Statistical significance is calculated using Student’s t test. Not significant (NS), ****p < 0.0001. [file Image_2.jpeg]

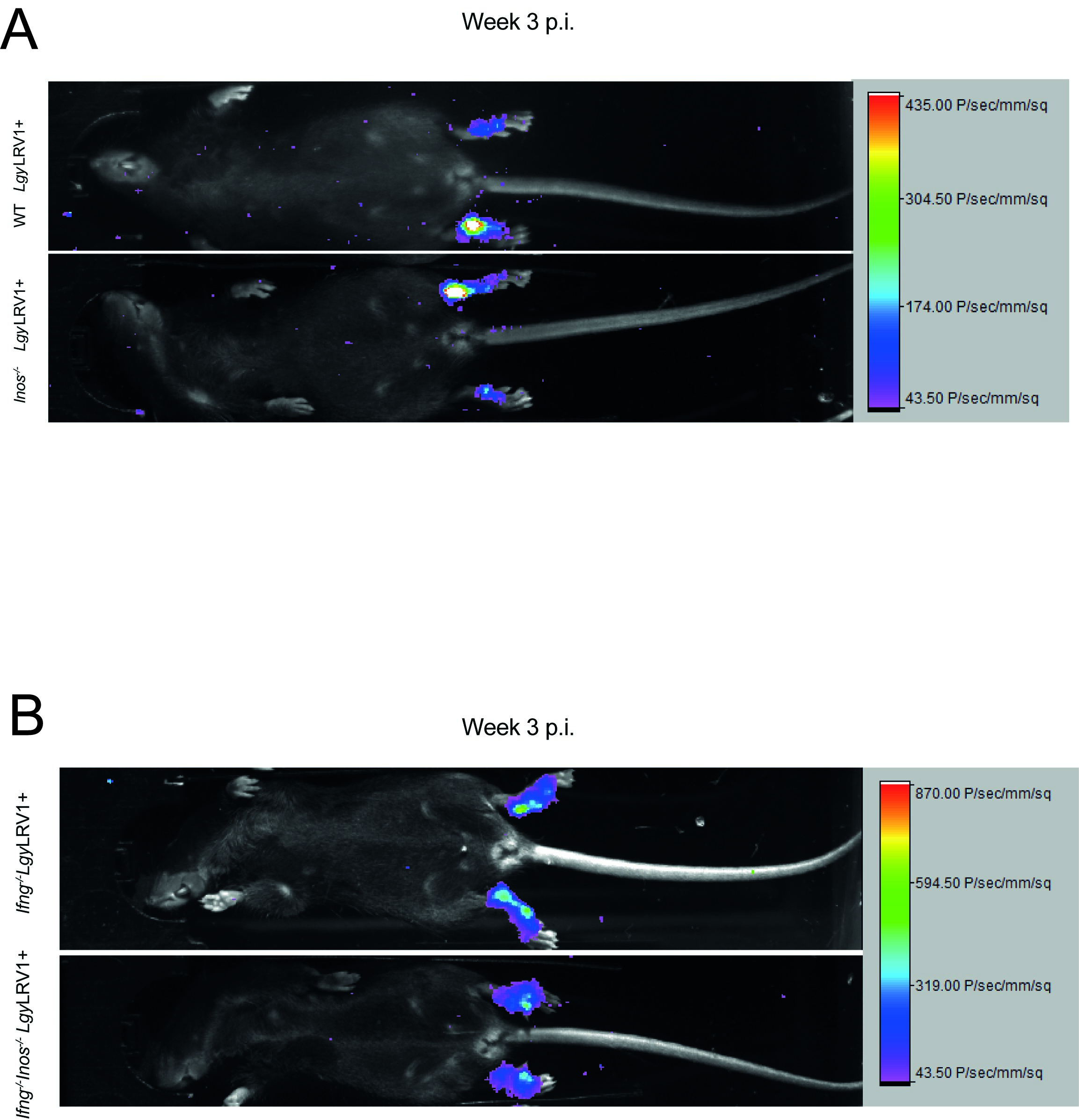

Supplement: Supplementary Figure 3 — Related to Figure 4 Ifng-/- and Ifng-/-Inos-/- mice display similar levels of parasitemia upon LgyLRV1+ infection. Hind footpads of (A) WT and Inos-/- and (B) Ifng-/- and Ifng-/-Inos-/- DKO mice were infected with 3 × 10^6^ stationary phase promastigotes of LgyLRV1+. Bioluminescence was quantified using Bruker Xtreme II as previously described (Reverte and Fasel, 2019). Representative images of bioluminescence are shown at the selected time point (3 weeks p.i.). [file Image_3.jpeg]

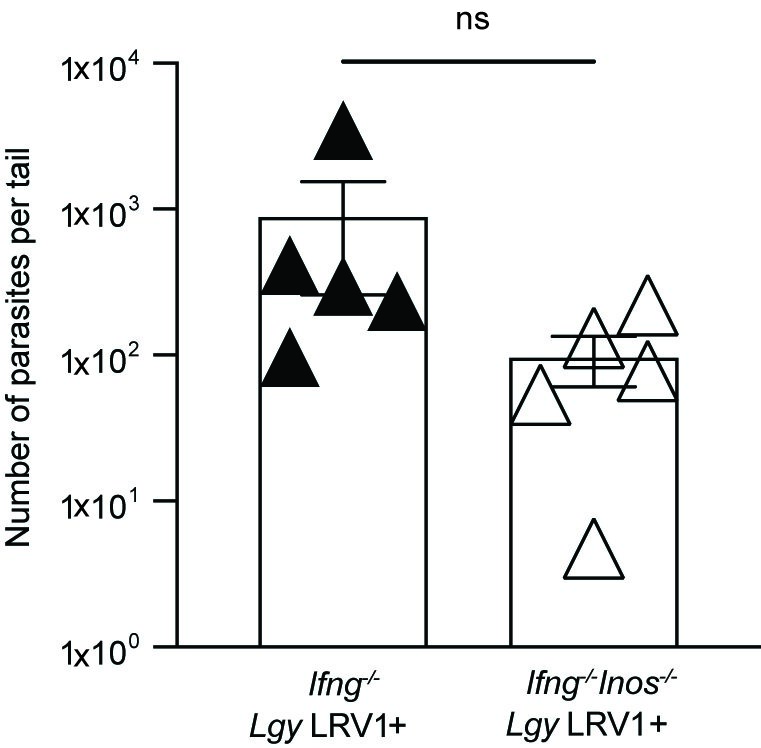

Supplement: Supplementary Figure 4 — Related to Figure 6 . Ifng-/-Inos-/- mice showed tendency for decreased levels of parasitemia in the tails compared to Ifng-/- upon LgyLRV1+ infection. Parasite burden at the tails was measured at week 8 p.i. by RT-qPCR, measuring Kmp11 gene expression. Data is representative from 2 independent experiments with 5 mice per group. Statistical significance is calculated using Student’s t test. Not significant (NS) [file Image_4.jpeg]
