# Supplementary material for: Leishmania guyanensis suppressed inducible nitric oxide synthase provoked by its viral endosymbiont
Source: Front Cell Infect Microbiol. 2022 Aug 12;12:944819. doi: 10.3389/fcimb.2022.944819 (PMC9416488; doi:10.3389/fcimb.2022.944819)
Supplement: Supplementary Table 1 — Oligonucleotides used in this study. [file Table_1.docx]

**Table 1. Oligonucleotides used in this study.**

| **Primer name** | **Sequence 5’-3’** |  |
| --- | --- | --- |
| **Primers for RT-qPCR** | |  |
| *Nos2* Fw | ACTACTACCAGATCGAGCC |  |
| *Nos2* Rev | ACCACTTTCACCAAGACTCTA |  |
| *Kmp-11* Fw | GCCTGGATGAGGAGTTCAACA |  |
| *Kmp-11* Rev | GTGCTCCTTCATCTCGGG |  |
| *L32* Fw | AAGCGAAACTGGCGGAAAC |  |
| *L32* Rev | TAACCGATGTTGGGCATCAG |  |
| **Primers for mice genotyping** | |  |
| *Ifng* Mutant Fw | CCTTCTATCGCC TTCTTGACG |  |
| *Ifng* WT Fw | AGAAGTAAGTGGAAGGGCCCAGAAG |  |
| *Ifng* Common | AGGGAAACTGGGAGAGGAGAAATAT |  |
| *Nos2* Mutant Rev | TCACCACCAGCAGTAGTTGC |  |
| *Nos2* WT Rev | TCCGATTTAGAGTCTTGGTGA |  |
| *Nos2* Common | CCTTCTATCGCCTTCTTGACG |  |
| *A20fl/fl sense* | CACAGAGCCTCAGTATCATGT |  |
| *A20fl/fl antisense* | CAGCGTGACCTGAGTTAGAAT |  |
| *A20fl/fl sense* | GCAGCTGGAATCTCTGAAATC |  |
| *LysM-Cre* Mutant | CTTGGGCTGCCAGAATTTCTC |  |
| *LysM-Cre* WT | TTACAGTCGGCCAGGCTGAC |  |
| *LysM-Cre* Common | CCCAGAAATGCCAGATTACG |  |
