# Supplementary material for: Leishmania guyanensis suppressed inducible nitric oxide synthase provoked by its viral endosymbiont
Source: Front Cell Infect Microbiol. 2022 Aug 12;12:944819. doi: 10.3389/fcimb.2022.944819 (PMC9416488; doi:10.3389/fcimb.2022.944819)
Supplement: Supplementary Table 2 — Antibodies used in this study [file Table_2.docx]

**Table 2. Antibodies used in this study**

|  |  |  |
| --- | --- | --- |
| **Antibodies** | **Source** | **Identifier** |
| Goat anti-mouse IgG (H+L) HRP Conjugate Antibody | Promega | Cat# W4021; |
|  |  | RRID: AB_430834 |
| Goat anti-rabbit IgG (H+L) HRP Conjugate Antibody | Promega | Cat# W4011; |
|  |  | RRID: AB_430833 |
| Rabbit Anti-iNOS Monoclonal Antibody | Cell Signaling | Cat# 13120S; |
|  |  | RRID: AB_944418 |
| Mouse Anti-γ-TUBULIN Monoclonal Antibody | Sigma-Aldrich | Cat# T5326; |
|  |  | RRID: AB_532292 |
| Rabbit Anti-Phospho STAT1 (S727) Ployclonal Antibody | Cell Signaling | Cat# 9177S; |
|  |  | RRID: AB_2197983 |
| Rabbit Anti-STAT1 Ployclonal Antibody | Cell Signaling | Cat# 9172; |
|  |  | RRID: AB_2198300 |
| Rabbit Anti-NF-kB B P65 Monoclonal Antibody | Cell Signaling | Cat# 8242; |
|  |  | RRID: AB_10859369 |
| Rabbit Anti-Phospho NF-kB B P65 (S536) Monoclonal Antibody | Cell Signaling | Cat# 3033; |
|  |  | RRID: AB_331284 |
| Rabbit Anti-A20/TNFAIP3 Monoclonal Antibody | Cell Signaling | Cat# 5630S; |
|  |  | RRID: AB_10698880 |
