# Supplementary material for: Leishmania guyanensis suppressed inducible nitric oxide synthase provoked by its viral endosymbiont
Source: Front Cell Infect Microbiol. 2022 Aug 12;12:944819. doi: 10.3389/fcimb.2022.944819 (PMC9416488; doi:10.3389/fcimb.2022.944819)
Supplement: Supplementary Table 3 — TLR agonists concentrations used in S1A, B Fig . [file Table_3.docx]

**Table 3. TLR agonists concentrations used in S1A, B Fig.**

| **Ligand:** | **FSL-1** | **Pam2CSK4** | **Pam3CSK4** | **LTA-SA** | **HKSA** | **HKSE** |
| --- | --- | --- | --- | --- | --- | --- |
| **Units:** | ng/ml | ng/ml | ng/ml | ng/ml | cells/ml | cells/ml |
| **Concentration:** | 10^1^ | 10^2^ | 10^2^ | 10^3^ | 10^7^ | 10^7^ |
| **Catalog No (Invivogen)** | tlrl-fsl | tlrl-pm2s-1 | tlrl-pms | tlrl-slta | tlrl-hksa | tlrl-hkse |
